# Environmental Predictors of Habitat Suitability for *Tetragonisca angustula* (Apidae: Meliponini) in the Andean–Amazonian Transition of Northern Peru

**DOI:** 10.3390/insects17090937

**Published:** 2026-09-07

**Authors:** Jhanet R. Puscan Inga, Jhonsy O. Silva-López, Elthon Hinojosa, Diego R. Salazar Ccatamayo, Hugo Frias Torres, Ligia Garcia, Julio Puscan-Rojas, Pável Matos Maraví, William Bardales, Jorge L. Maicelo-Quintana, Rainer Marco Lopez Lapa

**Affiliations:** 1 Laboratorio de Fisiología Molecular, Instituto de Investigación en Ganadería y Biotecnología, Facultad de Ingeniería Zootecnista, Biotecnología, Agronegocios y Ciencia de Datos, Universidad Nacional Toribio Rodríguez de Mendoza de Amazonas, Chachapoyas 01001, Peru; jhanet.puscan.epg@untrm.edu.pe (J.R.P.I.); elthon.hinojosa@gmail.com (E.H.); diego.salazar@unmsm.edu.pe (D.R.S.C.); hugo.frias@untrm.edu.pe (H.F.T.); william.bardales@untrm.edu.pe (W.B.); jmaicelo@untrm.edu.pe (J.L.M.-Q.); 2 Instituto de Investigación en Ganadería y Biotecnología, Facultad de Ingeniería Zootecnista, Biotecnología, Agronegocios y Ciencia de Datos, Universidad Nacional Toribio Rodríguez de Mendoza de Amazonas, Chachapoyas 01001, Peru; ligia.garcia@untrm.edu.pe; 3 Facultad de Ingeniería Zootecnista, Biotecnología, Agronegocios y Ciencia de Datos, Universidad Nacional Toribio Rodríguez de Mendoza de Amazonas, Chachapoyas 01001, Peru; 4 Área de Cartografía y Teledetección, Laboratorio de Agrostología, Instituto de Investigación en Ganadería y Biotecnología, Universidad Nacional Toribio Rodríguez de Mendoza de Amazonas, Chachapoyas 01001, Peru; 5 Instituto de Investigación para el Desarrollo Sustenible de la Ceja de Selva, Universidad Nacional Toribio Rodríguez de Mendoza de Amazonas, Chachapoyas 01001, Peru; 7278447092@untrm.edu.pe; 6 Biology Centre of the Czech Academy of Sciences, Institute of Entomology, 370 05 České Budějovice, Czech Republic; pavel.matos@entu.cas.cz

**Keywords:** MaxEnt, environmental suitability, forest cover, topographic heterogeneity, species distribution modeling, meliponiculture, Andean–Amazonian transition

## Abstract

The stingless bee *Tetragonisca angustula* (Apidae: Meliponini) is ecologically and socioeconomically important in northern Peru, yet the factors shaping its habitat suitability remain poorly quantified. We modeled current suitability across Amazonas and San Martín using 28 occurrence records: 17 field-georeferenced hive locations and 11 records from the Global Biodiversity Information Facility (GBIF). An initial set of 28 climatic, vegetation, hydrological, topographic, and edaphic predictors was reduced to 15 variables after repeated variance inflation factor analyses. Maximum Entropy models were calibrated with linear, quadratic, and hinge features, four regularization multipliers, 20 bootstrap replicates, and a 75:25 training–testing partition. A multiplier of 1.5 was selected for its higher minimum test AUC across replicates (0.72), indicating more consistent worst-case performance (training AUC = 0.94 ± 0.02; test AUC = 0.84 ± 0.07). Forest cover contributed most to model fit (31.7%), whereas predictions were most sensitive to slope (27.2% permutation importance), precipitation of the coldest quarter (14.2%) and soil organic carbon (13.3%). Suitability peaked at intermediate forest cover (~50%) and gentle slopes (~4°). Applying the 10th-percentile training-presence threshold, 1,190,582 ha (13.2%) were classified as suitable, concentrated in mixed forest–agricultural landscapes rather than continuous lowland forest. Vegetation structure and terrain, not climate alone, shaped predicted suitability; given uneven sampling, these patterns require field validation.

## Simple Summary

*Tetragonisca angustula* is a native stingless bee widely associated with tropical landscapes and traditional stingless-bee keeping in northern Peru. However, little is known about where environmental conditions are most favorable for this species across the Andean–Amazonian transition. We combined known hive and occurrence locations with information on climate, vegetation, terrain, water, and soils to identify suitable areas in Amazonas and San Martín. The results showed that suitable conditions were shaped by several environmental factors rather than climate alone, with forest cover and terrain playing particularly important roles. Approximately 13.2% of the study region was classified as suitable. However, only 3.11% of this predicted suitable habitat occurred within formally protected areas, while most was located in unprotected landscapes. These findings provide a spatial basis for future field surveys and can help guide conservation actions and the sustainable development of stingless-bee keeping, particularly in areas where suitable environments occur outside existing protected-area networks.

## 1. Introduction

Animal pollination plays a fundamental role in maintaining terrestrial ecosystem stability, enabling plant reproduction, and sustaining global agricultural production. Approximately 85% of angiosperm species depend, at least partially, on animal-mediated pollination, highlighting the ecological importance of pollinating organisms [1,2]. Nevertheless, habitat fragmentation, agricultural intensification, climate change, and pesticide exposure are accelerating biodiversity loss and altering pollinator communities worldwide [3,4,5]. Consequently, these interacting pressures may reduce pollinator diversity, disrupt plant–pollinator interactions, and weaken ecosystem resilience.

Within this broader context, wild bees contribute substantially to the reproduction of both native plants and cultivated crops, thereby supporting terrestrial biodiversity and food production [6,7]. Among them, stingless bees of the tribe Meliponini (Apidae: Meliponini) constitute a highly diverse group of eusocial insects distributed mainly throughout tropical and subtropical regions [8,9]. Current taxonomic assessments recognize more than 600 extant species, reflecting the considerable evolutionary and ecological diversity of this group [8]. In addition, several stingless bee species have high cultural and economic value because they are managed through traditional meliponiculture for honey, pollen, propolis, and pollination services [10,11].

Among Neotropical meliponines, *T. angustula* (Latreille, 1811) is widely recognized for its broad geographical distribution, eusocial organization, and flexible foraging behavior [12,13]. The species occurs in natural forests, agroecosystems, rural settlements, and urban environments, where colonies may occupy cavities in tree trunks, walls, roofs, and other human-made structures [14,15,16]. Furthermore, empirical studies indicate that *T. angustula* exploits floral resources from numerous plant families, allowing colonies to persist in landscapes with contrasting vegetation structures [17]. However, its apparent ecological flexibility does not imply that all environmental conditions are equally suitable for colony establishment and long-term persistence.

In particular, temperature, seasonal precipitation, vegetation complexity, nesting-site [18] availability, and agrochemical exposure may influence the foraging activity, reproductive performance, and survival of *T. angustula* colonies [19,20]. Thermal extremes can restrict flight activity, whereas changes in rainfall seasonality may alter flowering phenology and the temporal availability of nectar, pollen, and resin [19]. Likewise, habitat simplification can reduce the continuity of floral resources and suitable nesting cavities [21]. Therefore, the spatial distribution of the species is likely governed by the combined effects of climatic, topographic, edaphic, and land-cover conditions rather than by a single environmental factor.

These relationships are particularly relevant in the Andean–Amazonian transition of northern Peru, where the Amazonas and San Martín regions exhibit pronounced altitudinal, climatic, edaphic, and ecological gradients [22]. Their complex topography generates substantial spatial variation in temperature, precipitation, vegetation composition, and land use across relatively short geographical distances. As a result, environmentally suitable areas for *T. angustula* may be distributed discontinuously across valleys, montane forests, agroforestry systems, and lowland environments [23]. Moreover, sparse or geographically biased occurrence records may not adequately represent the full range of environmental conditions potentially available to the species [18]. To address these spatial limitations and evaluate geographic patterns of habitat quality, species distribution models have become fundamental analytical tools in modern biogeography and conservation.

Species distribution models provide a quantitative framework for relating georeferenced occurrence records to environmental predictors and estimating relative suitability across unsampled areas. Among presence–background approaches, Maximum Entropy modeling is frequently applied when reliable absence data are unavailable. Nevertheless, model performance depends strongly on the quality and spatial distribution of occurrence records, the definition of the calibration area, predictor selection, regularization, feature classes, and validation strategy [24,25]. Accordingly, model outputs should be interpreted as estimates of relative environmental suitability rather than direct representations of abundance, confirmed presence, or realized distribution.

Although recent studies have modeled the ecological niches of several stingless bee species, regionally calibrated assessments of *T. angustula* in northern Peru remain limited [12,26]. To address this knowledge gap, this study evaluates the current environmental suitability of *T. angustula* across the Amazonas and San Martín regions using presence–background modeling. Specifically, the objectives were to: (i) estimate current suitability by integrating bioclimatic, topographic, and edaphic predictors; (ii) identify the principal environmental variables associated with spatial suitability patterns; and (iii) quantify the extent of different suitability levels across both regions. The resulting information is intended to support future field surveys, pollinator conservation, and the spatial planning of sustainable meliponiculture.

## 2. Materials and Methods

### 2.1. Study Area

**Figure 1:**
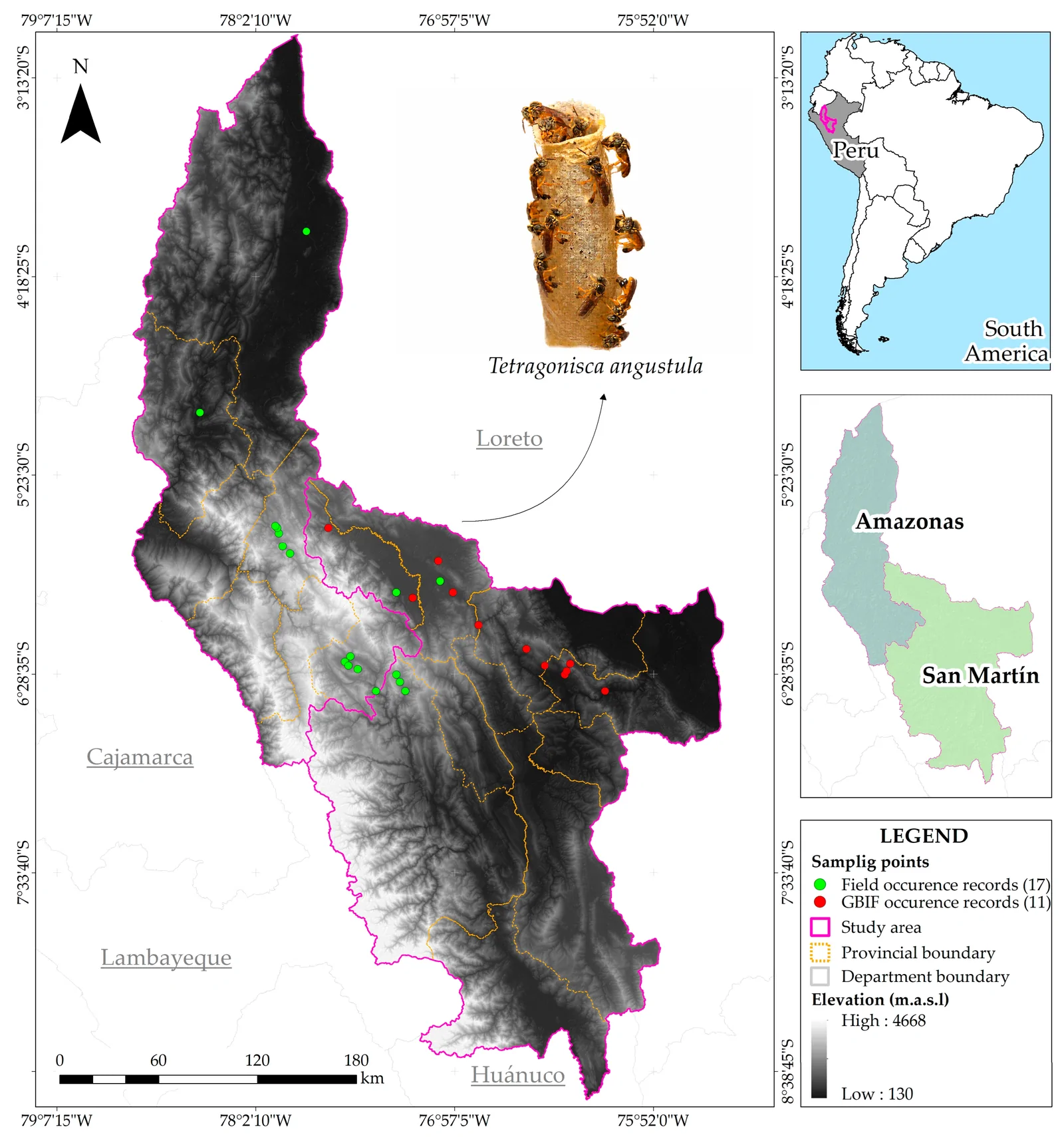
Study area and spatial distribution of *Tetragonisca angustula* occurrence records in Amazonas and San Martín, northern Peru. Green points indicate field occurrence records (17), and red points indicate GBIF occurrence records (11). The upper-right inset shows the location of Peru within South America, where the highlighted area indicates the country position. The lower-right inset shows the location of the study area within Peru, where the colored polygons represent the departments of Amazonas and San Martín, while the grey polygons represent the remaining national territory. Elevation is shown in meters above sea level.

The research was conducted within the adjacent departments of Amazonas and San Martín, northern Peru, covering a combined area of approximately 90,502 km^2^ [27,28]. Encompassing 17 provinces, the study region spans a significant portion of the Andean–Amazonian transition, characterized by an altitudinal range extending from Amazonian lowlands to high-Andean environments situated above 4300 m.a.s.l. [29]. This geographical heterogeneity generates pronounced spatial gradients in elevation, temperature, precipitation, and terrain complexity over relatively short distances [30,31]. Consequently, the study area comprises a diverse mosaic of lowland and montane forests, cloud forests, inter-Andean formations, and agricultural landscapes, such as coffee- and cacao-based agroforestry systems [32,33]. These land-cover categories, interspersed with remnants of native vegetation, exhibit variations in floral resource continuity and nesting-site availability, which critically influence the foraging activity and colony persistence of *Tetragonisca angustula* [34,35]. Within this heterogeneous spatial framework, we evaluated the current environmental suitability for the species; model outputs were interpreted as estimates of relative environmental suitability rather than as direct representations of confirmed presence, abundance, or realized distribution. The map of the collection points within the study area is shown in Figure 1.

### 2.2. Methodological Flowchart

**Figure 2:**
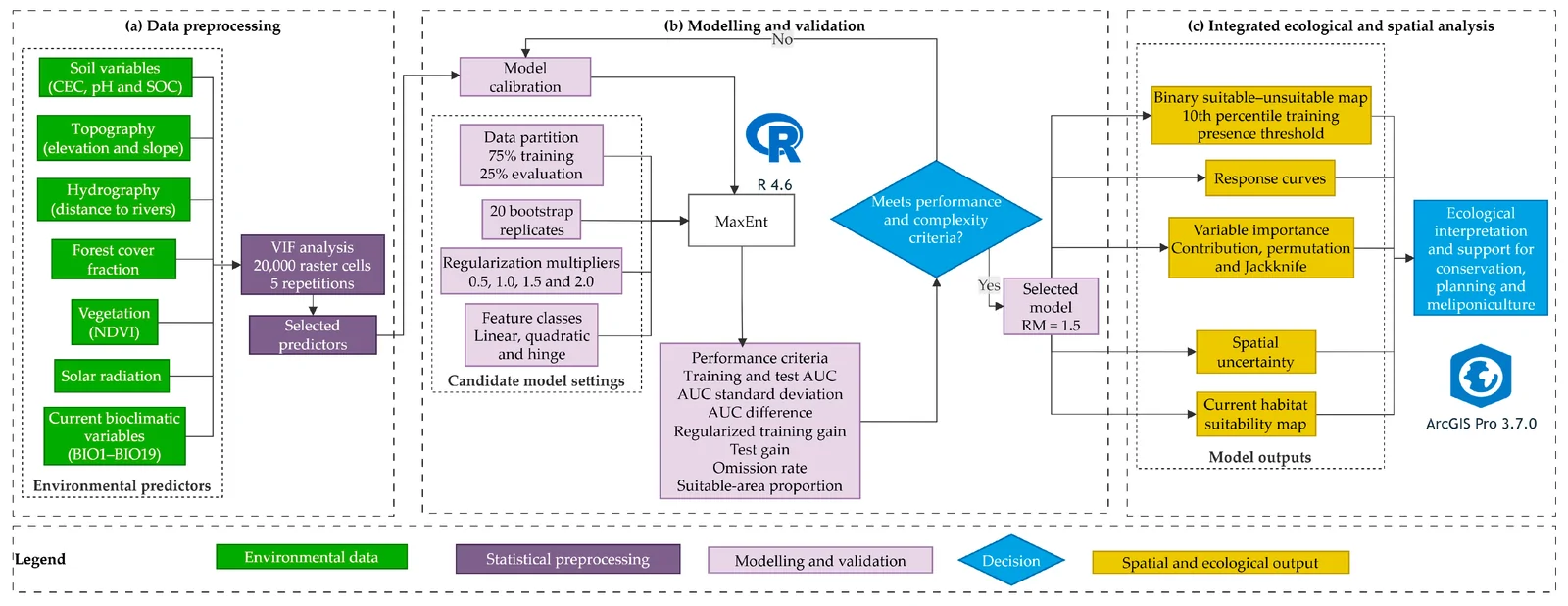
Methodological workflow used to estimate the current environmental suitability of *T. angustula* in Amazonas and San Martín, northern Peru. The workflow comprises three phases: (**a**) environmental-data preprocessing and predictor selection, (**b**) MaxEnt model calibration, tuning, and evaluation, and (**c**) generation and ecological interpretation of spatial outputs. The blue rectangle represents the final ecological interpretation and practical application of the model outputs to support conservation planning and sustainable meliponiculture. Abbreviations: AUC, Area Under the Receiver Operating Characteristic Curve; BIO1–BIO19, bioclimatic variables 1–19; CEC, cation exchange capacity; MaxEnt, Maximum Entropy; NDVI, Normalized Difference Vegetation Index; RM, regularization multiplier; SOC, soil organic carbon; VIF, Variance Inflation Factor.

Figure 2 illustrates the analytical workflow, structured into three sequential phases. The initial phase focused on the collation and refinement of occurrence records to ensure spatial and statistical robustness, as model accuracy is critically dependent on data quality, spatial distribution, and the minimization of sampling bias [18,24]. The environmental predictor set integrated the 19 standard bioclimatic variables with soil properties, specifically cation exchange capacity (CEC), soil pH, and soil organic carbon (SOC), alongside topographic variables, distance to rivers, forest-cover fraction, the Normalized Difference Vegetation Index (NDVI), and solar radiation; following spatial harmonization, potential predictor redundancy was evaluated through five independent Variance Inflation Factor analyses using 20,000 randomly sampled raster cells. In the subsequent modeling and validation phase, candidate Maximum Entropy models were calibrated in R version 4.6 using a 75:25 training–evaluation partition and 20 bootstrap replicates, with model complexity optimized by testing regularization multipliers of 0.5, 1.0, 1.5, and 2.0 in combination with linear, quadratic, and hinge feature classes. Acknowledging that model performance is substantially influenced by the calibration area and parameter tuning [25], configurations were systematically compared using the Area Under the Receiver Operating Characteristic Curve, AUC standard deviation, training and test gains, omission rates, and predicted suitable-area proportions. The configuration achieving the optimal balance between predictive performance and complexity (regularization multiplier = 1.5) was selected to generate the continuous environmental-suitability map, supplemented by a binary classification based on the 10th-percentile training-presence threshold, response curves, variable contribution scores, permutation importance, Jackknife results, and pixel-level spatial uncertainty, with all final cartographic outputs produced using ArcGIS Pro version 3.7.0.

### 2.3. Occurrence Records and Data Processing

The occurrence dataset comprised 28 records of *T. angustula*, including 17 hive locations georeferenced during field surveys using a Garmin Montana handheld GNSS receiver and 11 GBIF-mediated occurrence records. GBIF records were spatially filtered to retain only occurrences located within the Amazonas–San Martín study area and were retrieved from the Global Biodiversity Information Facility occurrence download (https://doi.org/10.15468/dl.jj3jdt, accessed on 5 August 2026). All occurrence coordinates were standardized in geographic coordinates referenced to the World Geodetic System 1984 before being incorporated into the species distribution modeling workflow.

### 2.4. Environmental and Soil Variables

The initial environmental dataset comprised 28 predictors representing the main climatic, vegetation, forest cover, hydrological, topographic, and edaphic gradients of the study area. Climatic conditions were characterized using 19 bioclimatic variables and solar radiation, while vegetation and forest conditions were represented by the Normalized Difference Vegetation Index (NDVI) and forest cover fraction. Topographic and edaphic conditions were represented by elevation, slope, cation exchange capacity (CEC), soil pH measured in water, and soil organic carbon (SOC).

Hydrological conditions were represented by the Euclidean distance from each raster cell to the nearest river or drainage channel, calculated from the hydrographic network provided by the National Water Authority of Peru. Elevation was obtained from the CGIAR-SRTM digital elevation model at 3 arc-seconds using the geodata package in R [36], and slope was subsequently derived from the elevation layer. The environmental datasets, variable codes, descriptions, and data sources are summarized in Table 1.

All environmental layers were projected to a common coordinate reference system and harmonized to a spatial resolution of 250 m. Spatial extent, cell size, pixel origin, and raster alignment were standardized to ensure spatial correspondence among predictors. The resulting environmental raster stack was subsequently used for multicollinearity assessment and predictor selection.

### 2.5. Multicollinearity Control

Multicollinearity among environmental predictors was assessed using the variance inflation factor (VIF), as strong linear dependence among variables can increase parameter uncertainty, reduce model efficiency, and complicate the interpretation of individual environmental effects [43,44]. A VIF threshold of 10 was adopted as a practical upper limit for problematic multicollinearity. This value was interpreted as an operational criterion rather than an absolute statistical rule and is consistent with its application in species distribution modeling studies [45,46].

Because VIF estimates derived from a single raster sample may depend on the selected cells, five independent random samples of 20,000 valid cells were drawn from the accessible area (M). In each repetition, the predictor with the highest VIF was iteratively removed until all remaining variables fell below the established threshold. Only predictors retained across all five repetitions were included in the consensus set. VIF values were then recalculated for this fixed set in each repetition and summarized as the mean ± one standard deviation.

Pearson correlation coefficients were calculated for the consensus predictors to describe their remaining pairwise relationships. Principal component analysis (PCA) was also performed using standardized predictor values to summarize the multivariate environmental structure of the selected dataset. All analyses were conducted in R 4.6.0 [47] using the usdm 2.1-7 [48] and terra 1.9-27 [36,49] packages.

### 2.6. Modeling with MaxEnt

The current potential distribution of *T. angustula* was estimated using MaxEnt version 3.4.4 through the maxent.jar graphical interface [50]. MaxEnt relates occurrence records to the environmental background available within the accessible area (M), allowing habitat suitability to be estimated without confirmed absence data. This presence-background approach is widely used in species distribution modeling when reliable absence information is unavailable [51,52].

Model calibration was conducted manually using linear, quadratic, and hinge feature classes, while product features and automatic feature selection were disabled. Four regularization multipliers were evaluated (0.5, 1.0, 1.5, and 2.0), with all remaining settings held constant. Each configuration included 20 bootstrap replicates, with 75% of the 28 occurrence records used for training and 25% reserved for testing. Models were fitted using 10,000 background points sampled within M, 5000 maximum iterations, a random seed, and cloglog output. Comparable MaxEnt studies have used similar data partitions, bootstrap procedures, background sampling, iteration limits, and regularization ranges [53,54].

Model discrimination was assessed using training and test AUC. For descriptive purposes, test AUC values were interpreted as excellent (>0.90), good (0.80–0.90), acceptable (0.70–0.80), poor (0.60–0.70), or uninformative (<0.60) [55]. Model selection also considered test AUC variability, the training–test AUC difference, regularized training gain, test gain, omission rates, the proportion of M predicted as suitable, and spatial agreement with known occurrences. These criteria were evaluated jointly because AUC alone does not fully describe predictive performance, model complexity, or the ecological plausibility of spatial predictions [56,57,58].

### 2.7. Threshold Selection and Overlay with Protected Areas

The mean cloglog output of the 20 bootstrap replicates was converted into a binary suitability map using the 10th percentile training presence threshold, computed directly on the mean surface from predicted values at the calibration records rather than by averaging replicate-specific thresholds. Cells at or above the threshold were classified as suitable. This criterion omits approximately 10% of the training occurrences with the lowest predicted suitability, reducing sensitivity to marginal conditions or spatial inaccuracies [59,60]. The maximum training sensitivity plus specificity threshold was additionally applied, and the resulting areas were compared [60].

Protected-area boundaries were obtained from the official SERNANP cadastre through the GEO ANP viewer (https://geo.sernanp.gob.pe/visorsernanp/, accessed on 5 August 2026; [61]). Four layers covered the three administrative tiers of the Peruvian system: nationally administered areas of the Sistema Nacional de Áreas Naturales Protegidas por el Estado (SINANPE); Reserved Zones, provisionally established but subject to the same legal provisions; Regional Conservation Areas (ACR); and Private Conservation Areas (ACP). Because conditions within reserves depend on the surrounding matrix, and tropical reserves are increasingly isolated by land-use change at their margins [62,63], peripheral zones were delineated as fixed-distance buffers of 1, 5 and 10 km with the source polygon subtracted. The shortest distance approximates the foraging range of *T. angustula* [64]; the wider ones characterise landscape context. Overlapping polygons were dissolved into a non-overlapping mask for regional totals, and buffers were clipped to exclude land within any other protected area.

The binary map was intersected with each protected area, each buffer ring, and the unprotected remainder. Because the study area spans UTM zones 17S and 18S, areas were computed on the WGS84 ellipsoid using cell-specific surface areas, which account for latitudinal variation in cell size, rather than by reprojecting to a planar system. For every protected area, intersected suitable habitat was expressed in hectares, square kilometers, and as a percentage of that reserve’s surface; equivalent totals were derived per tier, per department, and for the study region. Suitable areas inside reserves were classified as having formal conservation coverage, those outside as potential gaps. A representation ratio was obtained by dividing the proportion of suitable habitat within protected areas by the proportion of the study area under protection, with values below one indicating under-representation [65]. Mean forest cover was compared among reserves, buffers and unprotected land to assess whether suitability differences reflect the forest-cover gradient identified during calibration.

## 3. Results

### 3.1. Environmental Predictor Selection

Multicollinearity control retained 15 of the 28 initial environmental predictors: mean diurnal temperature range (BIO2), isothermality (BIO3), temperature seasonality (BIO4), maximum temperature of the warmest month (BIO5), precipitation seasonality (BIO15), precipitation of the warmest quarter (BIO18), precipitation of the coldest quarter (BIO19), solar radiation, NDVI, forest cover fraction, distance to rivers, slope, cation exchange capacity, soil pH, and soil organic carbon. These variables represented climatic, vegetation, hydrological, topographic, and edaphic gradients within the accessible area.

**Figure 3:**
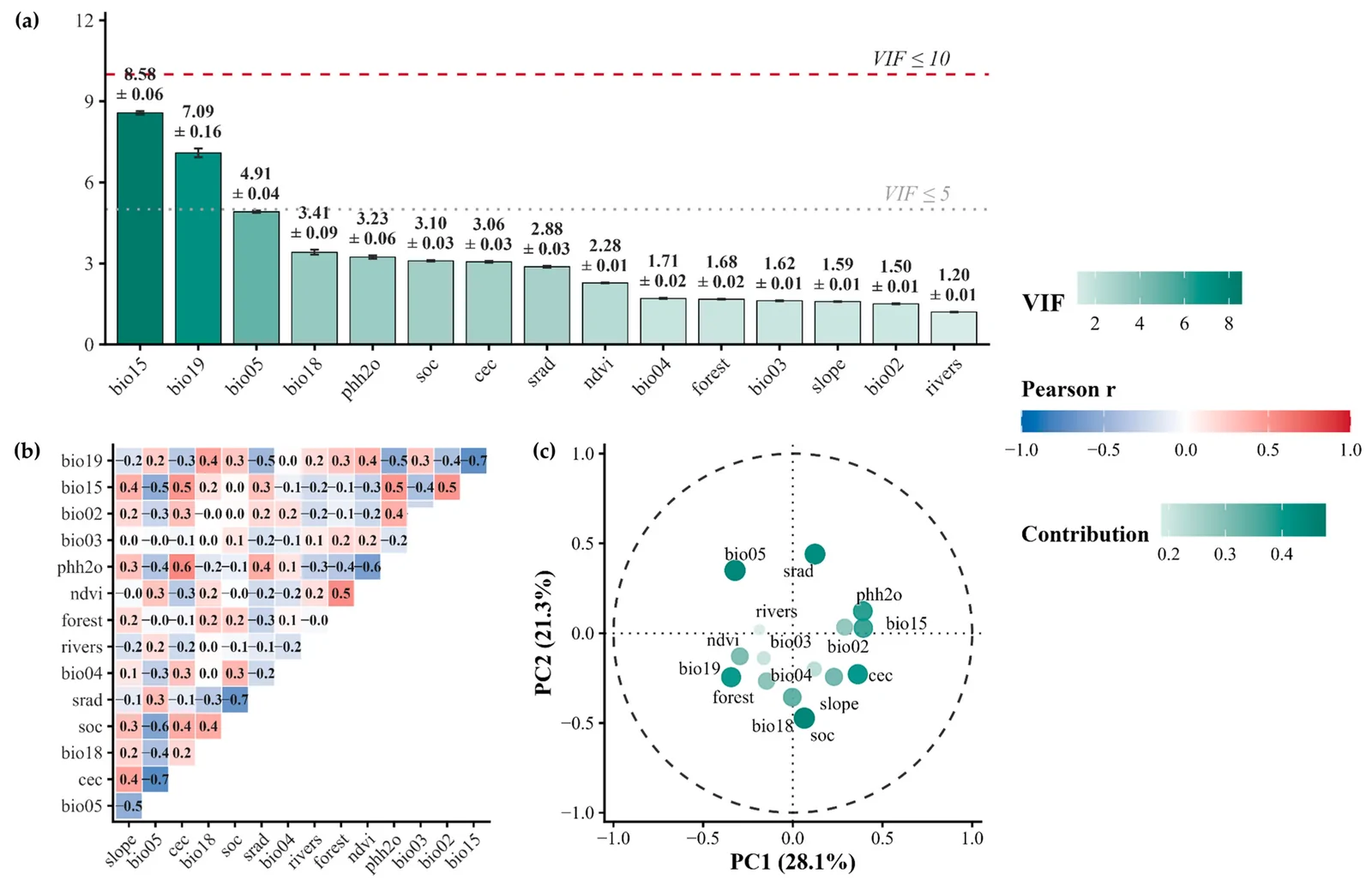
Multicollinearity and environmental structure of the selected predictors. (**a**) Mean variance inflation factor values and standard deviations across five repetitions; (**b**) Pearson correlation matrix; and (**c**) principal component analysis of the retained predictors.

Mean VIF values ranged from 1.19 to 8.80, and no value exceeded the established threshold of 10 (Figure 3a). Variation among the five repetitions was low, with a mean standard deviation of 0.071, a maximum standard deviation of 0.222, and a maximum individual VIF of 9.06; the minimum and maximum values recorded for each predictor are provided in Table S1. Only BIO15 and BIO19 exceeded the more conservative reference value of 5. The Pearson correlation matrix summarized the remaining pairwise relationships among predictors (Figure 3b), while principal component analysis showed that PC1 and PC2 explained 28.2% and 21.3% of the total variance, respectively, jointly accounting for 49.5% (Figure 3c).

### 3.2. Model Calibration and Selection

Model performance varied among the four regularization multipliers evaluated (Table S2). RM = 0.5 produced the highest training and test AUC values (0.9716 and 0.9201, respectively) and the lowest variability in test AUC (SD = 0.0550). However, it also showed the highest test omission rate (35.72%) and the most spatially restricted prediction, with only 4.61% of the accessible area (M) classified as suitable. RM = 1.0 exhibited the largest difference between training and test AUC (0.0678) and a test omission rate of 29.29%. Although RM = 2.0 produced the smallest training–test AUC difference (0.0497), it showed the greatest variability in test AUC (SD = 0.0750) and a lower test gain than RM = 1.5. In comparison, RM = 1.5 achieved training and test AUC values of 0.9321 and 0.8755, respectively, with a training–test AUC difference of 0.0566.

Its test omission rate was 20.72%, lower than those obtained with RM = 0.5 and RM = 1.0 and equal to that of RM = 2.0. RM = 1.5 also produced a higher minimum test AUC and test gain than RM = 1.0 and RM = 2.0, while its spatial prediction encompassed the known occurrences of *T. angustula* without generating the highly restricted distribution observed under RM = 0.5. Therefore, RM = 1.5 was selected because it provided the most appropriate balance among discrimination, omission, stability, model complexity, and spatial plausibility. Model selection was not based exclusively on the highest AUC, as this metric alone does not fully capture overfitting, predictive consistency, or the ecological plausibility of the resulting spatial pattern [57,58,66].

**Figure 4:**
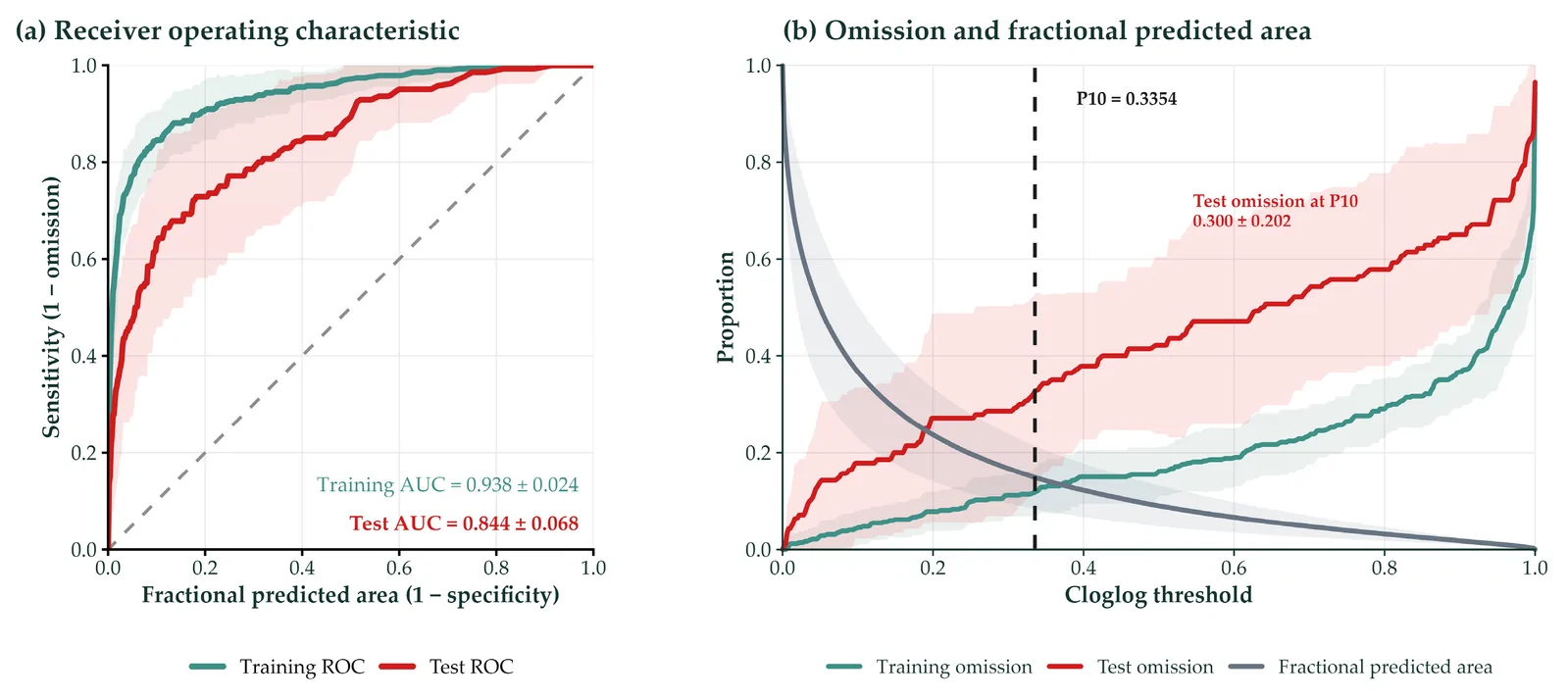
Predictive performance of the selected MaxEnt model. (**a**) Training and test ROC curves. (**b**) Training and test omission and fractional predicted area across *cloglog* thresholds. The vertical dashed line indicates P10, and shaded bands represent ± one standard deviation across 20 bootstrap replicates.

The predictive performance of the selected configuration was further examined using the mean ROC and omission curves obtained across the 20 bootstrap replicates (Figure 4). Mean training AUC was 0.938 ± 0.024, whereas mean test AUC was 0.844 ± 0.068, indicating good discriminatory performance for the withheld occurrence records, although test performance was lower and more variable than training performance. The mean 10th percentile training presence threshold was 0.3354. At this threshold, mean test omission was 0.300 ± 0.202, indicating considerable variability among bootstrap replicates.

### 3.3. Environmental Predictor Importance and Response Patterns

**Figure 5:**
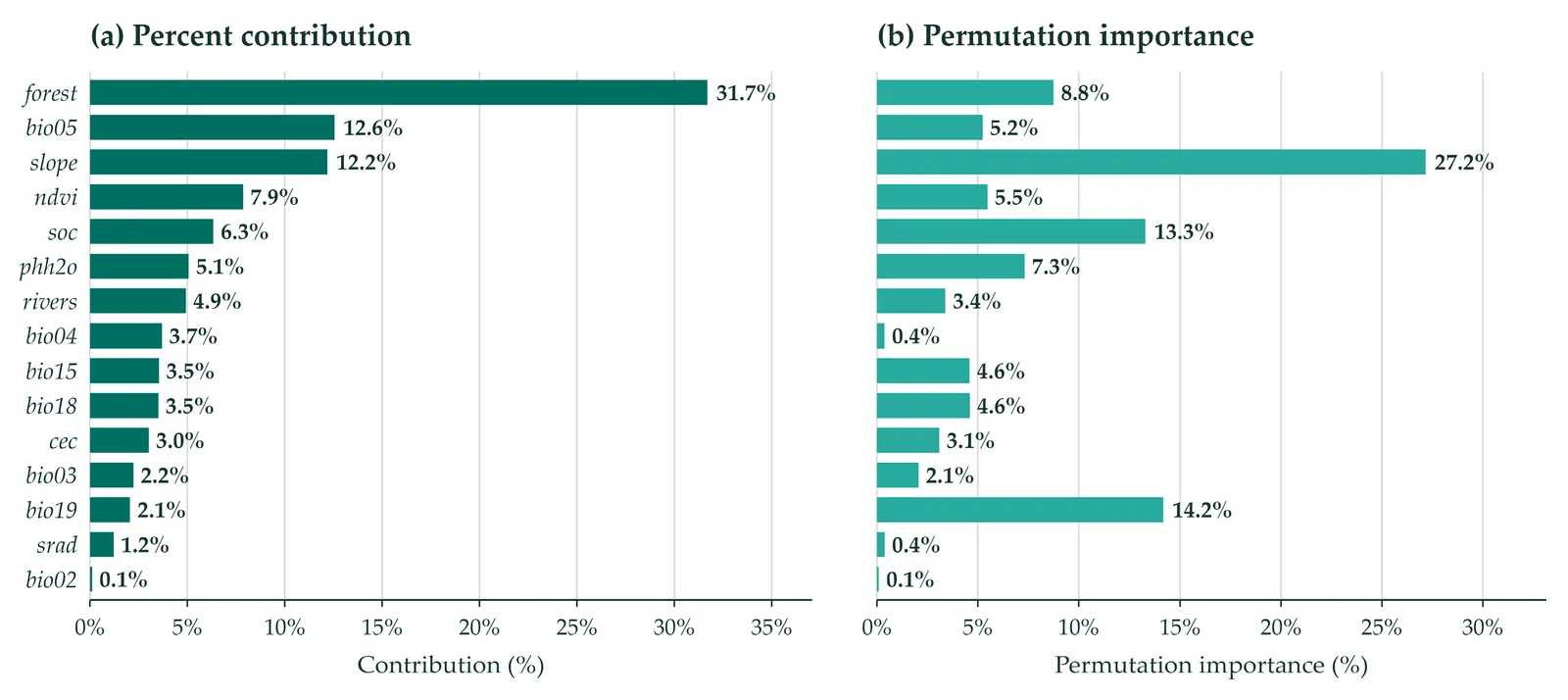
Environmental predictor importance in the selected MaxEnt model. (**a**) Percentage contribution and (**b**) permutation importance.

The relative influence of environmental predictors varied according to the importance metric considered. Forest cover showed the highest percent contribution (31.7%), followed by maximum temperature of the warmest month (BIO5; 12.6%), slope (12.2%), NDVI (7.9%), and soil organic carbon (6.3%). Together, these five predictors accounted for 70.7% of the total contribution. Soil pH and distance to rivers contributed an additional 5.1% and 4.9%, respectively, whereas each remaining predictor contributed less than 4% (Figure 5a).

Permutation importance produced a different ranking. Slope had the highest value (27.2%), followed by precipitation of the coldest quarter (BIO19; 14.2%), soil organic carbon (13.3%), forest cover (8.8%), and soil pH (7.3%). Together, these five predictors represented 70.8% of the total permutation importance. This contrast indicates that forest cover contributed most strongly during model fitting, whereas model predictions were particularly sensitive to changes in slope, BIO19, and soil organic carbon (Figure 5b).

**Figure 6:**
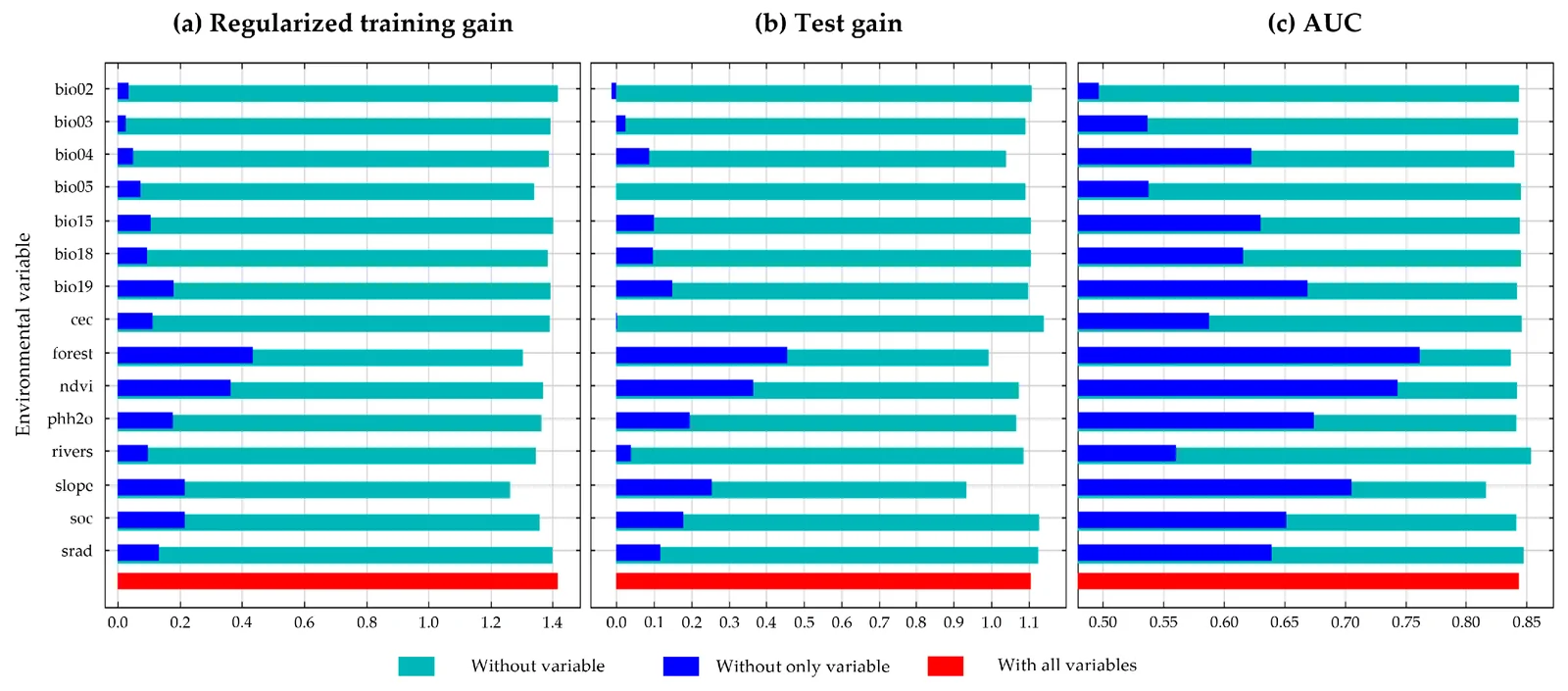
Jackknife analysis of environmental predictor importance in the selected MaxEnt model. (**a**) Regularized training gain, (**b**) test gain, and (**c**) area under the receiver operating characteristic curve (AUC). Turquoise bars indicate model performance when each predictor was omitted, blue bars indicate performance when each predictor was used independently, and red bars indicate performance when all predictors were included.

The Jackknife analysis provided complementary evidence on the independent and unique information contributed by each predictor. Forest cover produced the highest regularized training gain, test gain, and AUC when used independently, indicating the greatest individual predictive capacity. NDVI also showed comparatively high performance when evaluated independently. In contrast, omission of slope produced the largest decline in performance across the three Jackknife metrics, indicating that this predictor contained the greatest amount of unique information not captured by the remaining variables. Omission of forest cover also reduced model performance, confirming its complementary contribution to the final model (Figure 6).

**Figure 7:**
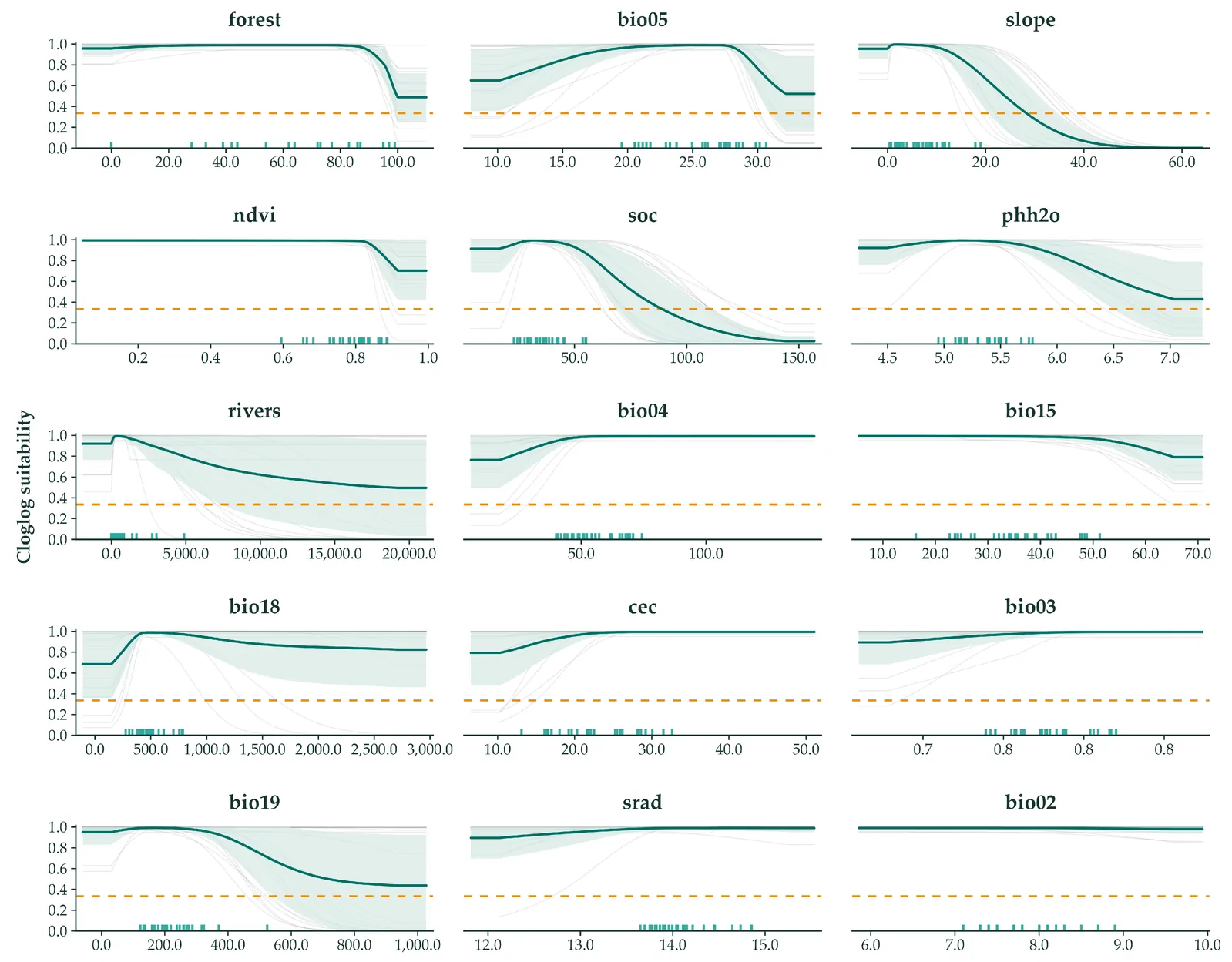
Marginal response curves of the environmental predictors in the selected MaxEnt model. Shaded bands represent variability among replicates, and labeled points indicate peak marginal suitability.

The marginal response curves showed predominantly nonlinear relationships between the environmental predictors and the estimated suitability for *T. angustula* (Figure 7). Peak suitability occurred at approximately 49.52 for forest cover, 24.73 °C for BIO5, 4.05° for slope, 0.43 for NDVI, 33.56 for soil organic carbon, and 5.20 for soil pH. For the remaining predictors, peak suitability was estimated at 591.88 for distance to rivers, 95.88 for BIO4, 24.22 for BIO15, 559.21 for BIO18, 37.09 for cation exchange capacity, 0.82 for BIO3, 179.62 for BIO19, 14.56 for solar radiation, and 7.66 for BIO2.

The response curves also showed contrasting functional patterns among predictors. Suitability remained high across broad ranges of forest cover, NDVI, BIO4, BIO15, BIO3, solar radiation, and BIO2, whereas sharper declines were observed for slope, soil organic carbon, soil pH, and BIO19 beyond their respective peak values. The shaded variability bands were wider for several predictors, particularly distance to rivers, BIO18, BIO19, and soil pH, indicating greater variation among model replicates. These model-derived peaks describe marginal suitability patterns and should not be interpreted as absolute ecological optima.

### 3.4. Provincial Distribution of Predicted Suitable Habitat

**Figure 8:**
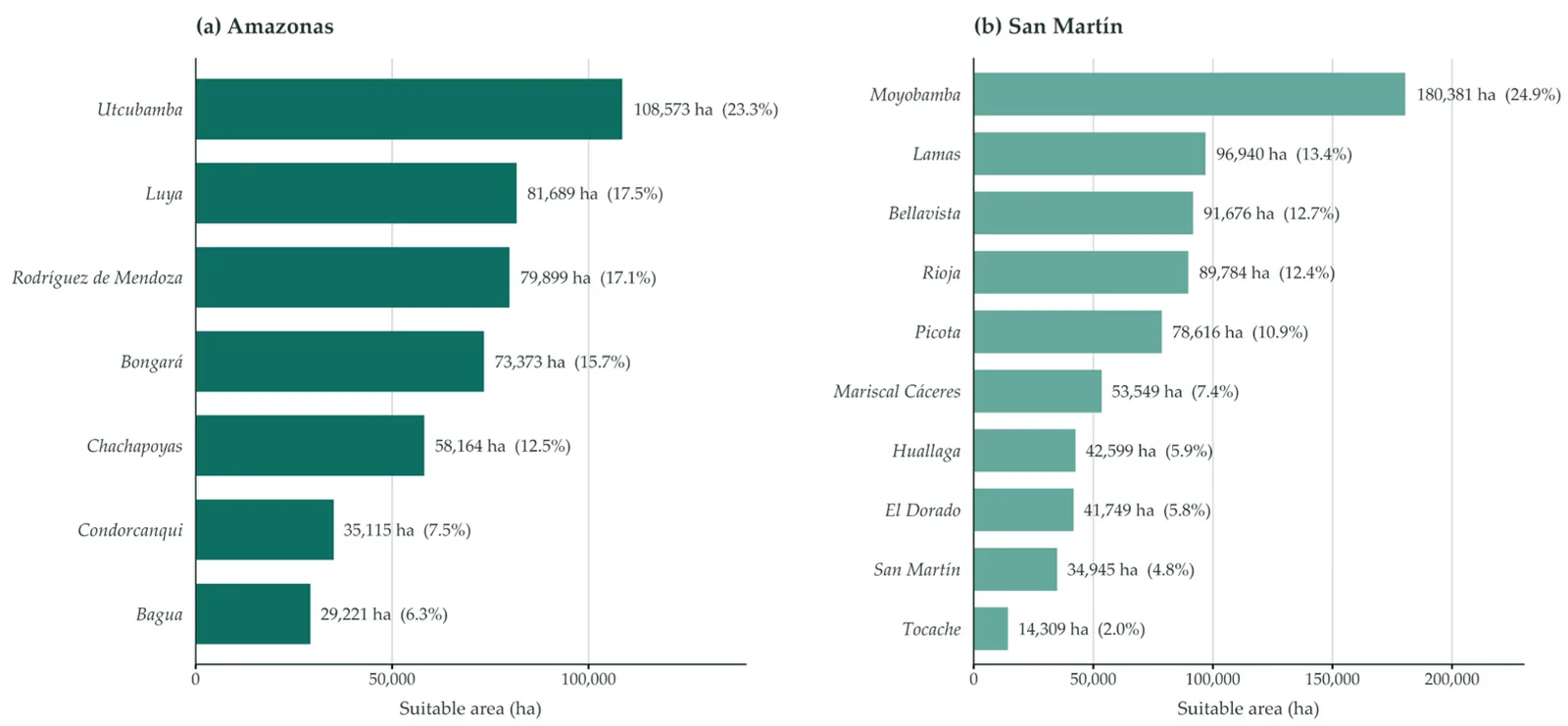
Provincial distribution of predicted suitable habitat. Suitable area in (**a**) Amazonas and (**b**) San Martín. Percentages indicate the contribution of each province to the total suitable area within its respective department.

In Amazonas, the predicted suitable habitat covered 466,032.67 ha. Utcubamba contained the largest suitable area, with 108,572.58 ha, representing 23.3% of the departmental total. Luya, Rodríguez de Mendoza, and Bongará followed with 81,689.19, 79,898.55, and 73,373.07 ha, respectively. The smallest suitable areas were identified in Condorcanqui and Bagua, with 35,114.69 and 29,220.95 ha, respectively (Figure 8a).

In San Martín, the predicted suitable habitat covered 724,549.55 ha. Moyobamba contained the largest suitable area, with 180,381.40 ha, equivalent to 24.9% of the departmental total. Lamas, Bellavista, Rioja, and Picota presented suitable areas ranging from 78,616.11 to 96,940.22 ha. The smallest suitable area was identified in Tocache, with 14,309.18 ha, representing 2.0% of the departmental total (Figure 8b). Detailed provincial estimates and the proportion of each province classified as suitable are provided (Table S3).

### 3.5. Representation of Suitable Habitat Within the Protected-Area Network

**Figure 9:**
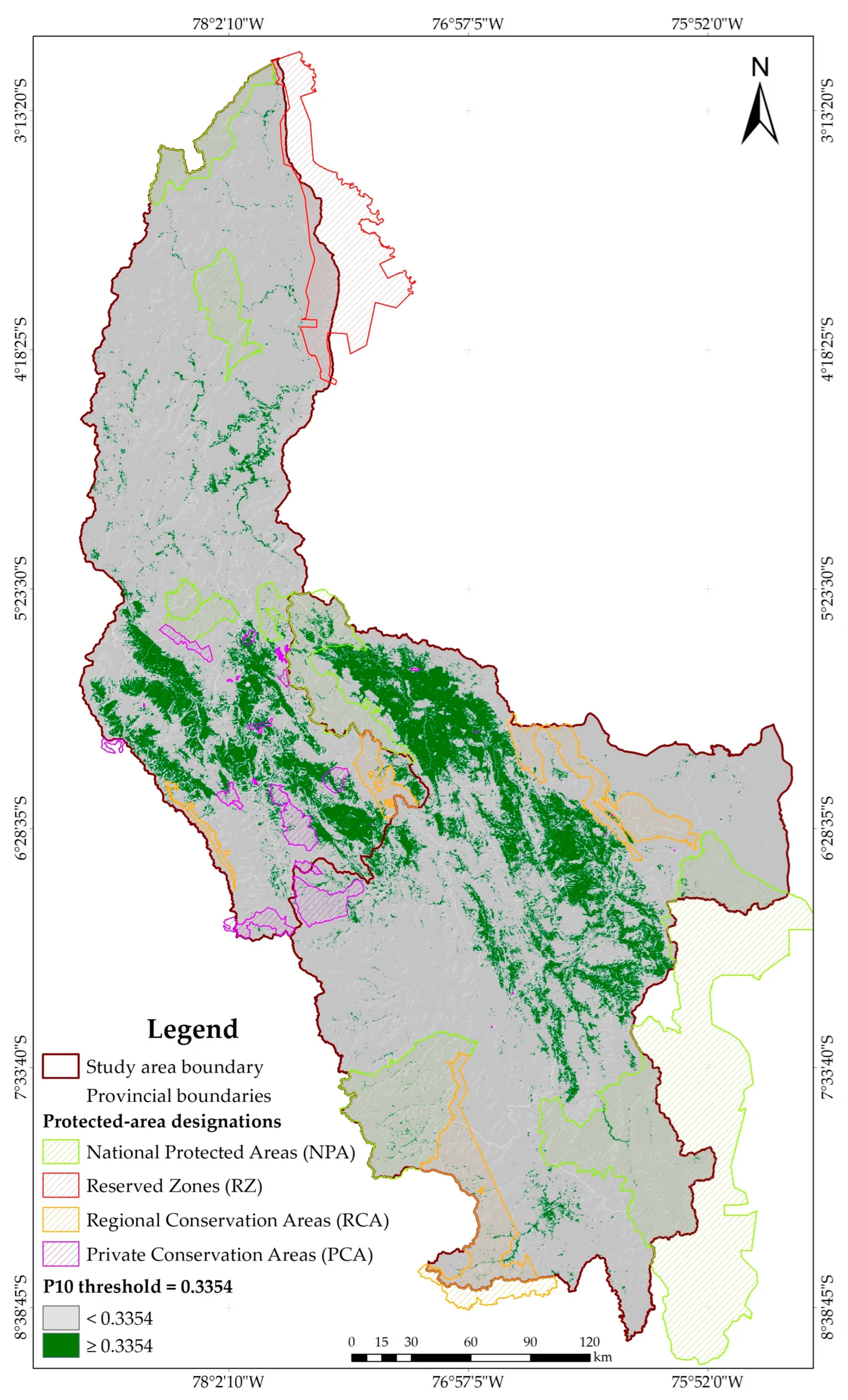
Spatial overlap between predicted suitable habitat for *T. angustula* and protected-area designations in Amazonas and San Martín, northern Peru.

As shown in Figure 9 and Table 2, protected areas covered 18,908.34 km^2^, equivalent to 20.92% of the study area. Of the 11,905.82 km^2^ classified as suitable for *T. angustula*, only 370.44 km^2^ occurred within protected areas, representing 3.11% of the total suitable habitat. In contrast, 11,535.38 km^2^, or 96.89% of the predicted suitable habitat, was located outside the protected-area network. Suitable habitat accounted for 1.96% of the total protected surface and 16.14% of the unprotected territory.

Among the conservation designations, National Protected Areas contained the largest absolute suitable area, with 211.55 km^2^, followed by Private Conservation Areas with 105.99 km^2^, Regional Conservation Areas with 51.29 km^2^, and Reserved Zones with 1.61 km^2^ (Table 2). However, Private Conservation Areas showed the highest proportion of their own extent classified as suitable (7.4%), compared with 1.7% in National Protected Areas, 1.3% in Regional Conservation Areas, and 0.1% in Reserved Zones.

## 4. Discussion

The present study shows that the predicted habitat suitability of *Tetragonisca angustula* across Amazonas and San Martín is associated with multiple environmental dimensions rather than being controlled exclusively by climate. Forest cover contributed most strongly during model fitting, whereas slope, precipitation of the coldest quarter, and soil organic carbon produced the highest permutation-importance values. This multidimensional pattern differs from broad-scale climate-only assessments of *T. angustula*, in which annual precipitation emerged as the dominant predictor, and suggests that regional models can reveal environmental associations that are obscured at continental scales [20]. Nevertheless, these predictors should be interpreted as spatial correlates of suitability rather than as experimentally demonstrated causal controls.

Forest cover was the most influential predictor during model fitting, supporting the ecological relevance of wooded environments for *T. angustula*. Vegetation cover may simultaneously represent the availability of floral resources, nesting cavities, plant resins, and buffered microclimatic conditions, although the raster used here cannot distinguish natural forest from agroforestry or other tree-dominated land covers. Previous studies have shown that vegetation structure and seasonal conditions influence floral-resource selection by *T. angustula*, while metabarcoding evidence indicates that stingless bees exploit a broad range of plants and frequently use forest-tree resources [19]. Therefore, the forest-cover response should be interpreted as an association with tree-dominated landscape structure rather than as direct evidence that every forested pixel constitutes suitable colony habitat.

Slope showed the highest permutation importance and contained the greatest amount of unique information according to the Jackknife analysis. Rather than representing a direct behavioral preference for steep terrain, slope probably integrates environmental variation in drainage, exposure, vegetation structure, and local climatic conditions across the Andean–Amazonian transition. The nonlinear response, with maximum predicted suitability at relatively gentle slopes, suggests that suitability may be concentrated in topographically heterogeneous but not extremely steep landscapes. Nevertheless, this interpretation requires caution because slope can covary with land use, accessibility, and the spatial distribution of sampled colonies.

Maximum temperature of the warmest month and precipitation of the coldest quarter indicate that seasonal and extreme climatic conditions remain relevant even though vegetation and terrain were more influential in the final model. Temperature can constrain the duration and timing of foraging, whereas seasonal precipitation may affect flowering dynamics and the temporal availability of pollen, nectar, and resin. Experimental observations of *T. angustula* showed that foragers experience more variable and warmer microclimates than nest workers and operate with lower warming tolerance than guard subcastes, indicating that external workers may be particularly sensitive to thermal stress [67]. However, response curves from correlative models describe associations within the calibration environment and should not be interpreted as physiological thresholds.

Soil organic carbon and soil pH contributed substantially to permutation importance, whereas cation exchange capacity and distance to rivers provided additional but smaller amounts of information. These variables are unlikely to affect adult bees directly at the mapped scale; instead, they may act as proxies for moisture conditions, vegetation composition, soil fertility, and the distribution of plant communities supplying floral and nesting resources. Stingless bees depend on plant-derived pollen, nectar, resins, and nesting substrates, linking their spatial ecology indirectly to environmental conditions that structure vegetation [8]. Accordingly, the edaphic associations should be interpreted as landscape-level correlations and not as experimentally established soil requirements of *T. angustula*.

Predicted suitable habitat was spatially heterogeneous across both departments, with a larger absolute area estimated in San Martín than in Amazonas. Nevertheless, absolute area alone does not indicate greater ecological suitability, because provincial and departmental extent strongly influences the number of suitable hectares. Recent field-based research in the Peruvian Amazon has also shown that *T. angustula* distributions are associated with elevation, nesting-tree availability, and exposure to deforestation, with more than half of the evaluated habitats overlapping areas of high forest-loss risk [68]. These findings reinforce the need to interpret the predicted suitability map jointly with forest condition, nesting-resource availability, and land-use pressure.

The protected-area analysis revealed a pronounced mismatch between where suitable habitat occurs and where formal protection has been established: only 3.11% of the predicted suitable habitat fell within the combined network of National Protected Areas, Reserved Zones, Regional Conservation Areas and Private Conservation Areas, leaving 96.89% on unprotected land, where suitable conditions were roughly eight times more frequent (16.14% versus 1.96%; representation ratio = 0.149). This is not an isolated result: modelling of 132 stingless bee species across the Brazilian Legal Amazon likewise found richness peaking outside protected areas and low representation within them, with *T. angustula* among the most widely recorded floral visitors [69], a convergence consistent with the tendency for reserves to occupy steep, high-elevation and remote terrain of limited agricultural value [70]—precisely the environmental space where our models predicted suitability to decline. A gradient within the network itself supports this reading, since Private Conservation Areas contained the highest proportion of suitable territory (7.41%), followed by National Protected Areas (1.72%), Regional Conservation Areas (1.26%) and Reserved Zones (0.15%), tracking landscape modification rather than the legal strength of the designation. Two cautions nonetheless apply: spatial overlap with a protected designation demonstrates neither effective management nor confirmed occupancy, and predicted suitability should not be equated with persistence, as a survey of 472 meliponiculturists documented mean annual colony losses of 43.4% across Latin America that rose with agricultural cover and declined with forest cover [71]. Conserving forest cover, nesting substrates and floral continuity outside protected boundaries—through agroforestry systems, riparian corridors and retention of nesting trees within farmland—therefore emerges as the more consequential intervention for *T. angustula*.

The selected MaxEnt configuration achieved useful discriminatory performance, but several sources of uncertainty must be considered. The model was calibrated with only 28 records, including 17 georeferenced hive locations and 11 GBIF occurrences, and the mean test omission at P10 was relatively high and variable among bootstrap replicates. In addition, the random 75:25 partition may not fully account for spatial autocorrelation, while managed-hive locations may partly reflect accessibility and decisions made by beekeepers rather than exclusively the environmental requirements of the species. Therefore, the outputs represent relative predicted suitability and should be validated through independent surveys, spatially structured evaluation, and separate analyses of wild, feral, and managed colonies.

## 5. Conclusions

This study provides a spatially explicit assessment of the current habitat suitability of *T. angustula* in the Amazonas Region of northern Peru using the MaxEnt modeling framework. Results indicate that environmental suitability is spatially restricted, with highly suitable habitats representing only a small fraction (5.8%) of the regional territory. Temperature-related variables, particularly temperature seasonality and minimum temperature of the coldest month, were identified as the primary drivers shaping the climatic niche of this species, highlighting the strong influence of thermal variability across Andean elevational gradients. Seasonal precipitation and edaphic conditions further contributed to explaining suitability patterns, emphasizing the importance of integrating multiple environmental dimensions when modeling pollinator distributions.

This study establishes a robust ecological baseline for understanding the climatic constraints shaping the distribution of *T. angustula* in northern Peru. By integrating climatic, topographic, and edaphic predictors at high spatial resolution, the model advances regional-scale pollinator biogeography in tropical mountain systems. The spatial restriction of highly suitable habitats highlights the need for proactive conservation planning and landscape-level management strategies to ensure the long-term sustainability of native stingless beekeeping systems.

## Figures and Tables

**Table 1:** Environmental datasets initially considered for species distribution modeling.

Environmental Dataset	Code	Brief Description	Data Source
Bioclimatic variables	BIO1–BIO19	Variables describing annual climatic trends, seasonality, and extreme or limiting temperature and precipitation conditions.	WorldClim v2.1 [37]
Solar radiation	srad	Mean incident solar radiation at the land surface, representing spatial variation in energy availability.	
Normalized Difference Vegetation Index	NDVI	Spectral vegetation index associated with vegetation greenness, photosynthetic activity, and canopy condition.	MODIS/Terra MOD13Q1 Collection 6.1 [38]
Forest cover fraction	forest	Percentage of each pixel covered by trees or forest vegetation, ranging from 0 to 100%.	Copernicus Global Land Service Land Cover 100 m, Collection 3 [39]
Hydrographic network	rivers	Euclidean distance from each raster cell to the nearest river or drainage channel.	National Water Authority of Peru [40]
Elevation	elevation	Terrain height above mean sea level.	Hole-filled CGIAR-SRTM v4.1 [41]
Slope	slope	Terrain inclination derived from elevation differences between neighboring cells.	
Cation exchange capacity	CEC	Soil capacity to retain and exchange positively charged ions.	SoilGrids 2.0 [42]
Soil pH in water	pH_2_O	Soil acidity or alkalinity measured in a water suspension.	
Soil organic carbon	soc	Amount or concentration of organic carbon stored in the soil.	

**Table 2:** Protected-area representation of predicted suitable habitat for *T. angustula* in Amazonas and San Martín, northern Peru.

Designation	Total (km^2^)	Suitable (km^2^)	% Desig.	% Regional
NPA	12,313.96	211.55	1.7	1.78
RZ	1093.27	1.61	0.1	0.01
RCA	4070.43	51.29	1.3	0.43
PCA	1430.69	105.99	7.4	0.89
All protected	18,908.34	370.44	2	3.11
Unprotected	71,461.63	11,535.38	16.14	96.89
Study area	90,369.97	11,905.82	13.2	100

## Data Availability

The original contributions presented in this study are included in the article thesis of Jhanet Rocio Puscan Inga, deposited in the institutional repository of the Universidad Nacional Toribio Rodríguez de Mendoza de Amazonas (https://repositorio.untrm.edu.pe/handle/20.500.14077/4952, accessed on 5 August 2026). Further inquiries can be directed to the corresponding authors.

## Supplementary Materials

The following supporting information can be downloaded at: https://doi.org/10.6084/m9.figshare.33158870, accessed on 5 August 2026.
